# Single-Stage vs. Multi-Stage Reconstruction in Multi-Ligament Knee Injuries: A Systematic Review and Meta-Analysis of Outcomes and Complications

**DOI:** 10.3390/jcm14196897

**Published:** 2025-09-29

**Authors:** Monketh Jaibaji, Omar Najim, Hamza Alali, Lisa Wood, Louw Van Niekerk, Tim Bonner, Andrea Volpin

**Affiliations:** 1 South Tees Hospitals NHS Foundation Trust, Middlesbrough TS4 3BW, UK; lisa.wood21@nhs.net (L.W.); louw.vanniekerk@nhs.net (L.V.N.); tim.bonner@nhs.net (T.B.); 2 Mid and South Essex NHS Foundation Trust, Chelmsford CM1 7ET, UK; onajim@icloud.com; 3 Faculty of Health Sciences, McMaster University, Hamilton, ON L8S 4L8, Canada; 1hamzaali2005@gmail.com

**Keywords:** single-stage, multi-stage, multi-ligament knee injuries, systematic review, meta-analysis, knee reconstruction

## Abstract

**Background/objectives:** Multi-ligament knee injuries (MLKIs) present complex surgical challenges, and there remains no consensus on whether single-stage or staged reconstruction yields superior outcomes. This study aimed to assess differences in complications, functional outcomes, and return-to-sport rates between single-stage and staged surgical approaches. **Materials and Methods:** A systematic review was conducted in accordance with PRISMA guidelines. Four databases (PubMed, Scopus, Embase, and the Cochrane Library) were searched for studies published between 2000 and 2025. Eligible studies reported surgical management of MLKIs and specified either single-stage or multi-stage reconstruction. Data on complications, functional scores (Lysholm), return to sport, rehabilitation protocols, and graft type were extracted and analyzed using descriptive statistics and study-level regression models. **Results:** A total of 43 studies encompassing 2086 patients were included (1900 single-stage; 186 multi-stage). Staged reconstruction was associated with a significantly lower rate of arthrofibrosis (1.95% vs. 7.29%; OR 3.96, *p* = 0.007), higher Lysholm scores (+4.7 points, *p* < 0.001), and higher return-to-sport rates (48% vs. 65%, *p* = 0.001) compared to single-stage. Use of synthetic grafts increased the risk of arthrofibrosis (OR 4.09, *p* = 0.031). Early mobilization and weightbearing were not associated with increased arthrofibrosis risk. **Conclusions:** Staged reconstruction may yield better functional outcomes and lower complication rates—particularly arthrofibrosis, compared to single-stage approaches. These findings support an individualized surgical strategy, guided by injury complexity, graft selection, rehabilitation goals, and patient-specific functional demands.

## 1. Introduction

Multi-ligament knee injuries (MLKIs) are complex injuries involving two or more major stabilizing structures in the knee, typically the anterior cruciate ligament (ACL), posterior cruciate ligament (PCL), medial collateral ligament (MCL), and lateral collateral ligament or posterolateral corner (LCL/PLC) [1]. These injuries most commonly result from high-energy trauma, although there is an increasing recognition of low-energy mechanisms, particularly in obese patients and sports-related contexts [2,3]. Given the potential for spontaneous reduction and missed diagnoses of disclocations, especially in lower-energy cases, the true incidence may be under-reported. Regardless, these are rare injuries, with epidemiological data from Australia showing incidences between 0.5 and 1.5 per 100,000, with a steady increase in incidence particularly amongst females aged 15–24. Rates amongst males are steadily declining [4], however, incidences overall remain higher amongst men. A large database study from the US estimated the incidences of knee dislocations to be notably higher at 3 per 100,000 trauma cases [5], which represents a steady increase from previous reported data [6]. Data specifically on multi-ligament knee injuries are more challenging to ascertain: registry data from Denmark between 2005 and 2017 showed 1160 MLKI surgeries performed, which is equivalent to 97 per year. This is compared to 28,843 isolated ACL reconstructions, suggesting that isolated ACL reconstructions are 25 times more common than MLKIs. However, this difference could be understated given the greater likelihood that ACL ruptures could be treated by conservative means [7]. Indeed, data from the US estimates that ACL reconstructions are up to 60 times more common than MLKIs [8]. Despite the low incidence, these remain devastating injuries with significant long-term consequences [9] and robust data regarding key clinical decisions such as the impact of staging are lacking.

Historically, surgical management has favored a staged approach. This typically involves initial repair of extra-articular structures followed by delayed reconstruction of the cruciate ligaments once range of motion has been restored. This strategy is thought to offer benefits such as improved rehabilitation control, optimized surgical timing, and a safer pathway for polytrauma patients with soft tissue compromise [10].

Recently, however, there has been a surge in interest in single-stage reconstruction, which brings into question the benefits of staged reconstruction and potentially allows for earlier return to full function and lower failure rates reported. There are also clear health economic benefits over multi-stage reconstruction. However, despite this surge, the literature directly comparing single vs. staged reconstruction specifically are lacking. In addition to this, while reviews on other aspects [11,12,13] of MLKI exist, no recent meta-analysis has compared the outcomes of single vs. staged reconstruction.

The primary objective of study was to compare the outcomes of single stage versus staged reconstruction in multi-ligament knee injuries focusing on complication rates, functional recovery, and return to sport (RTS). In addition, we sought to explore whether treatment level factors, such as graft type, rehabilitation strategy, and timing of surgery influence these outcomes. We hypothesized that staged reconstruction would be associated with (1) a lower risk of arthrofibrosis and graft failure, and (2) superior functional outcomes and return-to-sport rates compared with single-stage reconstruction.

## 2. Materials and Methods

A systematic review was conducted in accordance with the PRISMA guidelines (Preferred Reporting Items for Systematic Reviews and Meta-Analyses) [14]. Registration of the study and a study protocol was submitted to Prospero for approval (https://www.crd.york.ac.uk/prospero/ (accessed on 8 August 2025)). A comprehensive literature search was performed the first two authors across four databases, PubMed, Scopus, Embase, and the Cochrane Library, for relevant studies published between 2000 and 2025. The following search terms were used: “multi-ligament knee injury”, “MLKI”, “knee dislocation”, “knee ligament injuries”, “single-stage reconstruction”, “one-stage surgery”, “single operation”, “multi-stage reconstruction”, “two-stage surgery”, and “staged surgery”. Results were imported into Rayyan, an online-based systematic review management platform [15]. Reference lists of recent systematic reviews and included studies were also screened for any relevant publications.

**Figure 1:**
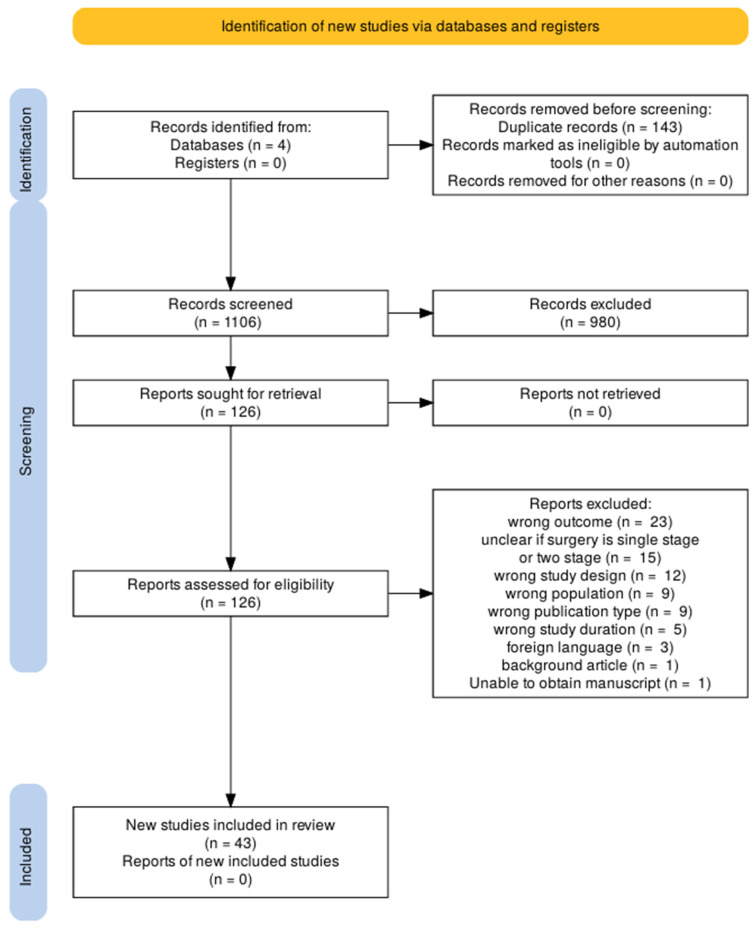
Studies included as per the PRISMA guidelines.

After removal of the duplicates, 1106 records remained. Abstracts were independently screened by the first three authors in accordance with the predefined inclusion criteria. A total of 126 full-text articles were retrieved, of which 43 met all eligibility criteria and were included in the final review. Reference lists of included studies were also screened for additional eligible studies, but none were identified. The PRISMA flowchart (Figure 1) summarizes the screening process.

Studies were eligible if they reported outcomes of single-stage and/or multi-stage reconstruction for MLKIs, had a minimum mean follow-up of 12 months, a mean patient age > 18 years, and involved at least two major knee ligaments. Inclusion also required reporting of at least one of the following: functional outcomes, return to activity, complication rates, or patient-reported outcome measures. Studies were excluded if single-stage and multi-stage outcomes were not reported separately, if the cohort involved only isolated ligament injuries or pediatric populations (<18 years old), or if the study was a case report or conference abstract without full data available. Studies where outcomes of single vs. staged reconstruction where not reported separately were excluded from quantitative synthesis. However, these studies were reviewed qualitatively and are discussed in the context of the prior literature.

Studies were grouped for synthesis based on the outcomes they reported. Only studies that directly compared single stage and staged reconstruction were included in the meta-analysis of functional scores. Studies reporting only one surgical approach were included in descriptive and regression analyses. Study-level characteristics (e.g., surgical timing, graft type, rehabilitation protocol) were reviewed to determine which studies contributed to each analysis, depending on whether they reported the relevant outcome measures.

Where necessary, summary statistics were converted to ensure consistency across studies. Means and standard deviations were calculated from medians and interquartile ranges using established methods when only non-parametric data were reported. Studies that did not report sufficient data for comparison were excluded from that specific synthesis but retained for narrative summary if relevant. No imputation was performed for missing outcome data.

All included studies were independently assessed for methodological quality using the MINORS (Methodological Index for Non-Randomized Studies) criteria by the first two authors M.J. and O.N. [7]. Any difference in opinion was referred to the senior author AV for final arbitration. The MINORS score produces a score out of 16 for non-comparative studies and out of 24 for comparative studies. For non-comparative studies (maximum score 16), we defined low risk of bias as a MINORS score of 13–16, moderate risk as 9–12, and high risk as ≤8. For comparative studies, low risk of bias (19–24 points), moderate risk of bias (13–18 points), or high risk of bias (≤12 points). No automated procedures were used in this process.

The certainty of evidence for each outcome was assessed using the GRADE (Grading of Recommendations, Assessment, Development and Evaluation) approach (https://www.gradeworkinggroup.org (accessed on 8 August 2025)), based on five domains: risk of bias, inconsistency, indirectness, imprecision, and reporting bias. Methodological quality was evaluated using the MINORS tool. GRADE assessments were performed separately for functional outcomes, complication rates, and return-to-sport data. As the number of directly comparable studies was limited, assessments of reporting bias and imprecision were made narratively rather than through formal statistical methods.

Results from individual studies were summarized in structured tables, including details on study design, sample size, surgical approach, follow-up duration, and MINORS scores. Functional outcomes and complication rates were tabulated by surgical stage (single vs. staged). Where comparative data were available, results were displayed using forest plots and box plots to visualize between-group differences. Meta-analyses and regression outputs were plotted and labeled with confidence intervals to facilitate interpretation of effect sizes across studies.

Data extraction was carried out by M.J., O.N. and H.A. The following variables were collected for each study: study design, year of publication, country of origin, total number of patients, mean age, percentage of male patients, ligaments injured, KD classification, time to surgery, surgical approach, graft type, rehabilitation protocol, functional scores, complications, return-to-sport rate, and follow-up duration.

A meta-analysis was performed for the subset of studies that directly compared single-stage and staged reconstruction and reported Lysholm scores. A random-effects model was used due to anticipated variation in surgical technique, rehabilitation protocols, and patient populations. Standardized mean differences (SMDs) with 95% confidence intervals were calculated to account for differences in scale and reporting. Heterogeneity was assessed using the τ^2^ statistic and further explored visually using box plots. The analysis was conducted in JASP (version 0.18.3; University of Amsterdam, Amsterdam, The Netherlands).

For outcomes not suitable for meta-analysis, descriptive statistics were used to summarize study-level characteristics. Continuous variables (e.g., Lysholm scores) were compared between groups using independent samples t-tests. Categorical variables (e.g., rehabilitation type, KD classification, graft type) were compared using chi-square or Fisher’s exact tests as appropriate.

Study-level binomial logistic regression, weighted by cohort size, was performed in R (version 4.3.1; R Foundation, Vienna, Austria) to examine associations between arthrofibrosis incidence and predictors including surgical timing, injury severity, graft type, and rehabilitation protocol. Functional outcome (Lysholm score) was assessed using univariate and multivariable linear regression models. For dichotomous outcomes such as complication rates (e.g., arthrofibrosis, infection, graft failure), we calculated odds ratios (ORs) with 95% confidence intervals (CIs). For continuous outcomes (e.g., Lysholm and IKDC scores), we used mean differences (MDs) when outcomes were measured using the same scale across studies and standardized mean differences (SMDs) when pooling across studies with similar constructs but different measurement scales. All effect estimates were reported with 95% confidence intervals. These effect measures were selected to allow consistent interpretation of treatment effects across included studies. All regression analyses were conducted in R. Descriptive and exploratory data review was conducted in JASP (version 0.18.3; University of Amsterdam, Amsterdam, The Netherlands). A two-sided *p*-value of <0.05 was considered statistically significant. These analyses also served to explore potential sources of heterogeneity across studies, including variation in surgical staging, graft choice, timing of surgery, and rehabilitation protocol. No formal sensitivity analyses were performed due to the limited number of comparable studies and the overall low methodological quality of the included studies.

## 3. Results

### 3.1. Study Characteristics

A total of 43 studies with 2086 patients were included in this review. Table 1 and Table 2 display the study characteristics of the studies that have comprised this review. Across all studies, 1900 patients had single-stage procedures whereas 186 patients had staged procedures. The mean age is 32.9 years weighted across all studies (32.7 single-stage, 35.1 multi-stage).

Mean follow-up was at 62.8 months ± 42.1 (range: 12–182 months). In total, 30% of studies reported outcomes beyond 5 years. Multi-stage procedures demonstrated longer average surveillance periods (71.6 months) compared to single-stage approaches (52.1 months).

Acute repairs, described as surgery < 3 weeks post-injury, encompassed 697 patients over 15 studies, with a mean delay of 1–3 weeks. On the other hand, chronic repairs (>3 weeks post-injury) were reported in 29 studies, with delays ranging from 6 weeks to 3+ years. The longest delays were in Gupta et al. [42], which had PCL repairs at 115 weeks, and Pizza et al. (2023), which had repairs at a mean of 3.3 years [35].

The mean time to surgery was around 12.5 weeks (median 6 weeks) across studies reporting exact data. Single-stage surgeries tended to be performed earlier (4–8 weeks) compared to staged approaches, with time between surgeries often being over 6 weeks.

### 3.2. Single- vs. Staged Reconstruction

**Figure 2:**
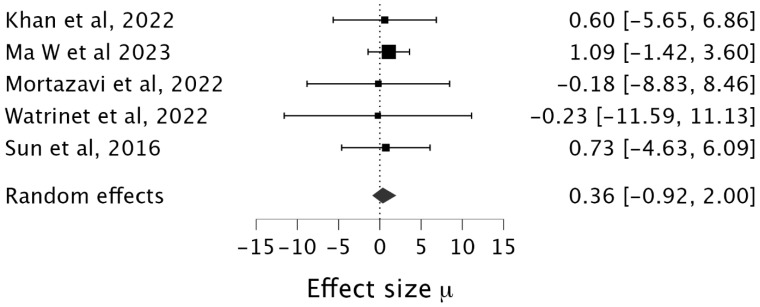
Box plot of a meta-analysis of 5 studies [16,17,18,19,20].

Only five studies directly compared single- and staged outcomes. A meta-analysis of five studies (n = 206 patients) was performed. Single-stage reconstruction showed a marginal advantage in Lysholm scores (standardized mean difference [SMD] = 0.36, 95% CI [−0.03, 2.00]), but this did not reach clinical or statistical significance; see Figure 2. Moderate between-study variation (τ = 0.39, 95% CI [0.04, 1.39]), demonstrated in Figure 2, likely reflects differences in surgical technique and rehabilitation protocols. There were some notable technical differences between single-stage and staged cases, for example, autografts (hamstring tendon) were predominant in single-stage studies, while allografts/synthetics were used in staged cohorts. Single-stage procedures were performed earlier than staged (mean 3.2 weeks post-injury vs. 6.8 weeks for multi-stage).

### 3.3. Functional Outcomes

When conducting an analysis of all studies, we found a significant heterogeneity in the functional outcomes used in across all the studies. The Lysholm (31 studies) and the international knee documentation committee subjective form (IKDC) scores (20 studies) were the most utilized outcome measures. An independent sample t test demonstrated that the final Lysholm scores were significantly better in the staged cohort (n = 136) compared to the single-stage (n = 1636) [82.47 ± 11.32 vs. 87.21 ± 7.65, mean difference: −4.74 [6.33, −3.15], *p* < 0.001]. However, there was no significant difference when comparing the IKDC scores at final follow-up [76.31 ± 15.25 vs. 77.88 ± 9.45, MD −1.57 [−4.23, 1.10], *p* = 0.19); see Table 3. In multivariate linear regression, delayed surgery was independently associated with a significantly lower Lysholm score (β = −9.99, *p* = 0.013), and increasing age was also negatively associated with outcome (β = −0.72, *p* = 0.047). Surgical stage (single- vs. Staged), KD severity, and sex were not significant predictors (R^2^ = 0.256, *p* = 0.088).

### 3.4. Complication Rates

The most reported complications across all studies were arthrofibrosis, graft failure, and infection. Across all studies the risk of arthrofibrosis was significantly higher in single-stage compared to multi-stage at 7.29% vs. 1.95%, OR 3.96 (95% CI 1.25–12.57) *p* < 0.05. Graft failure tended to be higher in single-stage surgery; however this did not reach statistical significance, 3.08% vs. 0.65% OR 4.85 (95% CI 0.67–35.31), *p* = 0.125. Infection rate was higher in multi-stage cohorts, 0.84% vs. 1.95% 0.42 (95% CI 0.12–1.49), *p* = 0.167, see Table 4.

A study-level binomial logistic regression weighted by cohort size was performed to explore factors associated with arthrofibrosis risk. Predictive variables included early mobilization, early weightbearing, presence of high-grade knee dislocations (KD-IV to KD-V), and surgical staging (single- vs. staged reconstruction). Early mobilization demonstrated a non-significant trend toward increased risk (OR 1.36; 95% CI: 0.92–2.01; *p* = 0.126), while early weightbearing showed no effect (OR 0.97; 95% CI: 0.67–1.41; *p* = 0.885). Staged surgery did not significantly differ from single-stage in arthrofibrosis risk (OR 1.40; 95% CI: 0.93–2.13; *p* = 0.108). With respect to classification of injuries, KDI-III injuries were associated with a significantly lower risk of arthrofibrosis (OR 0.50; 95% CI: 0.29–0.86; *p* = 0.012). The use of synthetic grafts was associated with a significantly higher risk of arthrofibrosis compared to allografts (OR 4.09; 95% CI: 1.14–14.72; *p* = 0.031).

### 3.5. Rehabilitation and Return to Sport

Functional outcomes were compared across studies using mean Lysholm scores in relation to postoperative rehabilitation protocols. At the study level, there was no difference in Lysholm scores between early and delayed mobilization groups (82.7 ± 8.0 vs. 82.7 ± 10.7; *p* = 0.996). However, studies that reported early weightbearing showed a trend toward higher functional scores, though this did not reach the threshold statistical significance (85.7 ± 6.6 vs. 80.4 ± 10.6, *p* = 0.068). In a multivariable linear regression model adjusting for age, sex, and injury severity, neither early mobilization nor early weightbearing was significantly associated with Lysholm score. Early mobilization demonstrated a negligible effect (β = −0.92; 95% CI: −9.49 to 7.65; *p* = 0.824), while early weightbearing showed a non-significant positive trend (β = +2.66; 95% CI: −5.94 to 11.26; *p* = 0.524).

Return-to-sport (RTS) data were available across 18 studies. Overall, patients undergoing staged reconstruction demonstrated a significantly higher return to sport rate compared to those undergoing single-stage procedures, where a reported 44/53 (84%) patients treated with a staged approach returned to sport compared to 238/516 (46%) patients treated with single-stage surgery, *p* = 0.029.

### 3.6. Acute vs. Delayed Surgery

Between the earlier vs. delayed groups, there was no difference in IKDC scoring MD −1.49 (SE = 7.89), with no statistically significant difference observed (*p* = 0.86). The mean difference in Lysholm scoring in the acute and delayed groups was −0.49, with a small, non-significant effect size (Cohen’s d = −0.07, SE = 0.63), and no statistically significant difference (*p* = 0.908). With respect to complications, arthrofibrosis was more likely with early surgery (OR: 1.72, 95% CI = [0.97, 3.03] *p* = 0.061); however, this was not statistically significant. Analysis of eight studies showed significantly higher odds of graft failure in early 27/164 vs. 28/312, OR 2 [95% 1.13, 3.52], *p*-value = 0.023. Patients undergoing acute surgery had a significantly higher return to sport rate compared to delayed surgery (95.2% vs. 74.0%, *p* < 0.001).

### 3.7. Risk of Bias Assessment

Most of the studies were judged to be of moderate to high risk of bias based on the MINORS criteria; see Table 2. Of the five comparative studies the median MINORS score was 17/24, interquartile range (IQR) 16–18. In the remaining non-comparative studies the median score was 12/16 (IQR 10–13). Using the GRADE framework, the certainty of evidence was rated as low for functional outcomes and very low for complication rates and return to sport. While most included studies contributed meaningful clinical data, the overall body of evidence was limited by retrospective designs, variable reporting quality, and small sample sizes in several comparisons. Inconsistencies in outcome definitions and follow-up reporting were noted, particularly for complications and return to sport. As only a subset of studies directly compared surgical staging, publication bias could not be formally assessed but was considered possible. Thus, for functional outcomes the certainty was rated as low according to the GRADE framework. For complication rates and return-to-sport outcomes, the certainty was limited. These findings should therefore be interpreted with caution but still provide meaningful insight into trends across the current literature.

## 4. Discussion

To our knowledge, this is the largest review to date focusing specifically on outcomes of single versus staged reconstruction in multi-ligament knee injuries (MLKIs). Notably, most studies in this review focused on single-stage reconstruction, potentially reflecting a shift in contemporary surgical practice. Indeed a recent consensus statement reported that most experts agree that single-stage management should be attempted where possible [59]. However, they note the lack of robust evidence currently in the literature. Functional outcomes appeared to favor staged reconstruction, although the difference was only statistically significant in Lysholm scores. Lysholm scoring focuses more on symptom severity [60] while IKDC scores focus more on overall function [61]. Staged reconstruction may offer more controlled rehabilitation and reduced soft tissue trauma per operative episode, potentially translating into improved range of motion. However, these benefits did not consistently lead to superior functional outcomes.

Our analysis found significantly higher rates of postoperative stiffness in single-stage surgery. However, in our multivariate regression analysis, surgical staging itself was not a statistically significant, independent predictor of arthrofibrosis. Mook et al. [11] demonstrated improved range of motion in their systematic review comparing single vs. staged reconstruction of MLKIs. Notably they only examined the most severe MLKI, involving both cruciate ligaments and both collaterals. Our analysis included a broader spectrum of injury severity and in our model, KD classification was the strongest predictor of arthrofibrosis risk.

The association between the timing of surgery and stiffness risk remains controversial. Ozbek [13] et al. in a recent meta-analysis found that surgery within three weeks significantly increased the risk of postoperative stiffness though earlier systematic reviews and numerous large multicenter studies have disputed this, with Hohmann et al. finding superior outcomes in early surgery [62]. Marder et al. [63] in a review of 31 studies concluded that there was insufficient evidence to establish any difference in outcomes between early and delayed surgery, and Vermeijden et al. [64] concluded no difference in outcomes. This discrepancy may be explained by heterogeneity in injury severity and patient selection. It is plausible that staging is more important than timing in higher-grade injuries, where soft tissue compromise and external fixator use necessitate a delayed or phased approach.

Our analysis found no significant association between early mobilization and improved functional scores. Importantly, high-grade injuries (KD IV and V) were most strongly associated with arthrofibrosis risk, reinforcing the notion that injury severity, rather than timing or staging, may be the dominant determinant of postoperative stiffness. Ozbek et al. [13] found that the risk of postoperative stiffness was significantly lower when two ligaments were injured compared to three. This raises the question of patient selection and whether KD III injuries (involving three ligaments) are better managed with a single-stage or staged procedure. Other factors may also influence this decision, particularly the presence of concomitant injuries in high-energy trauma. The degree of soft tissue compromise can often necessitate delayed surgery, as can the use of an external fixator to span the knee. Two studies have specifically investigated the use of external fixators in MLKI outcomes. Bi et al. [65] found external fixator use to be independently associated with an increased risk of requiring manipulation under anesthesia (MUA) following reconstruction. In contrast, Hanley et al. [66] did not identify an independent association between external fixator use and stiffness; rather, their findings highlighted injury severity as the key determinant. Currently, there is no consensus on the optimal duration of external fixator use, and in practice, this is likely influenced by patient-specific factors and institutional resource constraints. The use of synthetic grafts was significantly associated with an increased risk of arthrofibrosis. Synthetic grafts have previously been linked with inferior outcomes compared to autografts in knee ligament reconstruction [67], suggesting their use should be avoided where possible. In many cases, their use likely reflects the necessity imposed by severe or complex injuries.

The effect of staged surgery on graft failure has also been previous studied in the literature. Our analysis showed that single-stage surgery tended to have higher rates of graft failure than staged reconstruction, 3.08% vs. 0.65%, OR 4.85 (95% CI 0.67–35.31. The effect of staged surgery on graft failure has also been previously examined in the literature. Our analysis showed that single-stage reconstruction was associated with a higher rate of graft failure compared to staged procedures (3.08% vs. 0.65%; OR 4.85, 95% CI 0.67–35.31). LaPrade et al. [57] reported excellent functional outcomes following early single-stage reconstruction in sports-related MLKIs. They proposed that early restoration of normal knee kinematics may reduce the risk of graft failure. However, single-stage reconstruction may present technical challenges related to optimal graft tensioning. The simultaneous reconstruction of multiple ligaments introduces competing force vectors, which may predispose to suboptimal tensioning and eventual failure [68]. In addition, soft tissue swelling in the acute setting may compromise tunnel positioning accuracy [69] and increase the risk of tunnel convergence [70]. Optimal ligament tensioning is also key to avoiding stiffness [71]. Regardless of surgical staging, the overall risk of graft failure observed in our review was lower than reported in some large single-center studies. Mussell et al. [28], for example, reported an ACL graft failure rate of 8% in a cohort composed primarily of athletic patients. This suggests that graft failure may be underreported in the broader literature. The higher physical demands placed on grafts in athletic populations may also partly explain the increased failure rate observed in that study.

Among studies that reported surgical technique details (see Table 2), there was substantial variability in both the ligaments reconstructed and the order of graft tensioning. Most commonly, reconstruction involved the ACL, PCL, MCL/PMC, and PLC/LCL in various combinations, with the PCL frequently addressed first in tensioning sequences. In several studies, the cruciate ligaments were tensioned prior to the collateral structures, while others prioritized repairing the collateral ligaments first. However, reporting on this was inconsistent, and nearly half of the studies did not specify the sequence of graft tensioning or ligament order, limiting the ability to draw definitive conclusions regarding best practice.

PCL fixation first is the most widely reported technique as the central axis of rotation of the knee and the main stabilizer [2]. This has been supported by early biomechanical studies such as Moashe et al. [72], who concluded that while both PCL and ACL first improved rotational and sagittal alignment, ACL tensioning first was more likely to result in anterior tibial translation. They also drew firm conclusions against PLC tensioning first as it excessively increased internal rotation of the tibia. More recent data on porcine knees by Zheng et al. [73] concluded that ACL tensioning at 30 degrees first restored normal knee kinematics in flexion compared to PCL tensioning. This approach does raise concerns about the risk of elongation and failure of the PCL graft. Kim et al. [68] describes a simultaneous tensioning technique of both ACL and PCL. In their small retrospective study, they found better posterior laxity in the simultaneous tensioning cohort but no difference in anterior laxity. They also found better functional outcomes in their simultaneous tensioning group. Conclusions should be limited due to low numbers and the retrospective nature of the study; however, it does provide clinical evidence that graft tensioning sequences is relevant, and optimization of sequencing protocols can mitigate some of the theoretical concerns of single-stage surgery. This underscores the need for future studies to report technical details such as graft fixation order, as variations may confound comparisons between single and staged strategies.

No study stratified outcomes based on the number of ligaments reconstructed. The majority of two-stage studies reported the reconstruction/repair of the PCL and collaterals in stage 1 and the ACL was left for stage 2. We also found considerable variation in how collateral ligaments were managed, including differences in surgical repair versus reconstruction and the conservative management of extra-articular ligament injuries. The lack of standardization in ligament reconstruction order and collateral management may partly explain the heterogeneity observed between single and staged approaches and highlights the importance of reporting these details in future studies.

Rehabilitation in our analysis did not show a statistically significant effect on outcomes, though early weightbearing did show a positive trend. Rehab protocols were generally poorly described across studies. Three studies [24,28,57] highlighted the importance of early ROM as a means of reducing postoperative stiffness and promoting earlier return to sport. Their focus on athletes suggests a highly motivated patient cohort with access to tailored rehab protocols and perhaps is not applicable to the general population. Most patients in these cohorts had lower-grade knee dislocations, which are associated with better outcomes irrespective of surgical staging or timing. Any effort to introduce early mobilization protocols must carefully balance the potential benefits with the increased risk of graft failure and the need for strict patient compliance.

Other factors have also been investigated in relation to MLKI outcomes. Dean et al. [3] examined the difference in outcomes between high-energy and low-energy injury mechanisms. Interestingly, they found no significant difference in Lysholm or IKDC scores between the two groups. However, patients with low-energy mechanisms had higher Tegner activity scores. This may reflect the inclusion of sports-related injuries in the low-energy cohort, potentially accounting for the increased postoperative activity levels.

The study also highlighted variability in how high-energy and low-energy mechanisms were defined across the literature. Furthermore, other potentially influential factors such as patient comorbidities or injury context were not consistently analyzed, limiting broader conclusions. This also raises an important issue regarding classification on MLKIs, we found significant differences in the way injuries are classified, this has been noted in the literature [59] and reflects a number of issues. First is the limitations of current classification systems in adequately describing the injury; second, we found that MLKI and dislocation were on occasion used interchangeably; however, the majority MLKIs result in dislocation [8]. The presence of knee dislocation grossly impacts [9,74] outcomes, thus reporting of the data needs to reflect this. Poplosky et al. [75] created a pathoanatomic classification for MLKI combing elements of the Schenk and KD classifications. This is arguably the most anatomically distinct classification system currently available and its use going forward may help in the stratification of outcomes in MLKIs. A notable limitation is the lack of accounting of non-ligamentous qualifiers such as neurovascular injury, loss of joint congruence or presence of meniscal tears. Despite this, the adoption of a universally agreed classification system would represent significant progress.

Return to sport (RTS) is a key outcome, particularly in the athletic population. Across the included studies, we found significant variability in reported RTS rates, ranging from 39% to 100%. Staged reconstruction demonstrated higher RTS rates compared to single-stage procedures (84% vs. 46%, *p* < 0.001); however, this finding should be interpreted with caution due to the substantially smaller sample size in the staged cohort.

Everhart et al. [76], in a cohort of 524 patients, reported an overall RTS rate of 54%. A more recent review by D’Ambrosi et al. [12] found a 75% RTS rate at any level, although this dropped to 60% among elite athletes. More recent data from Borque et al. [77] reported an 88% return to elite-level sport at a minimum two-year follow-up. Whether staged reconstruction offers a meaningful advantage for patients aiming to return to sport remains unclear, as outcomes are often confounded by the need for earlier recovery timelines, particularly among elite athletes.

The effect of the body mass index has also been studied with varying results. High BMI individuals are at an increased risk of knee dislocation via low energy mechanisms [78]. Numerous studies have found that obese patients consistently demonstrate inferior functional outcomes and higher failure rates compared to non-obese cohorts [79,80,81]. However, there has been no consistent association with postoperative stiffness, arthrofibrosis, or the requirement of a manipulation under anesthesia [65].

Only 30% of the studies included in this review had a mean follow-up duration greater than five years. This is significant, as Klasen et al. [82] demonstrated in a recent meta-analysis that outcomes following MLKI reconstruction tend to decline substantially over the long term, particularly in PCL-based injuries, highlighting the need for more extended follow-up studies. Boos et al. [33], with a mean follow-up of 5.2 years, reported an 11% late conversion rate to total knee arthroplasty, further emphasizing the importance of long-term surveillance. Given the relative rarity of MLKIs, conducting high-quality randomized controlled trials or large-scale long-term studies remains challenging. National registries, such as the Swedish National Ligament Registry, may serve as valuable tools in capturing long-term outcome data and guiding future clinical decision-making.

There are several limitations to this study. First, many included papers were of low methodological quality (MINORS score < 12), and the majority were retrospective in design. Only five studies directly compared single-stage and staged reconstruction, and none were randomized controlled trials. Substantial heterogeneity was observed in Lysholm scores (τ = 0.8), likely due to variability in surgical techniques, rehabilitation protocols, patient selection, and inconsistency in injury classification systems. No current consensus exists on the optimal classification system and functional outcome scores in MLKI. Additionally, reporting of complications was often poor, thus complication rates are potentially underestimated. We also reported on studies from across the world with variable access to resources including synthetic grafts and rehabilitation services. Health economics also may also influence the decision to undertake single or staged surgery, however reporting on this is limited. The certainty of these findings was therefore limited and thus the results should be interpreted with caution. Studies reporting on staged reconstructions generally had small sample sizes, further limiting statistical power. As previously discussed, the lack of long-term follow-up across most studies prevents accurate assessment of delayed complications such as reoperation rates. The review was also limited by the authors’ inability to access some non-English language publications, reducing the number of MLKI cases included. Finally, there is no universally accepted definitions for key terms such as stiffness, graft failure, or complications, making inter-study comparisons more difficult. However, these limitations are reflective of the general limitations in research into MLKIs and are common to all reviews on this topic. This systematic review and meta-analysis does put forward clinically meaningful data that can aid in the decision-making process and highlights gaps in the knowledge base. We also propose areas of future research and data collection that can help build a more robust evidence base.

## 5. Conclusions

In conclusion, multi-ligament knee injuries (MLKIs) are complex and challenging to treat, with numerous variables influencing patient outcomes. While staged reconstruction appears to be associated with better functional results and a lower risk of arthrofibrosis and graft failure, our findings indicate that multiple factors such injury severity contribute significantly determinant of prognosis than surgical staging. The heterogeneity in patient presentations, injury patterns, and associated complications highlights the importance of avoiding a one-size-fits-all approach. Ultimately, our study supports the need for an individualized, patient-specific strategy to optimize outcomes for those with MLKIs, considering injury grade, overall health, and rehabilitation potential. Based on current evidence including the results of this review, we advocate the consideration of a staged approach in higher-grade injuries.

## Figures and Tables

**Table 1:** Study characteristics comprised in the review.

Study Characteristic	*n*
Study design	
Retrospective case series	37
Prospective studies	5
Comparative studies	3
Mixed retrospective and prospective	1
Geographic distribution	
USA	10
China	8
India	7
Europe	10
Other	6
Publication year	
2020–2024	23
2010–2020	14
2000–2010	6

**Table 2:** Summary of studies.

Study ID	Study Design	Total Patients (N)	Single-Stage (n)	Staged (n)	Follow-Up (Months)	MINORS Score	Ligaments Reconstructed/Repaired	Sequence of Tensioning
Khan et al., 2022 [16]	Retrospective comparative study	27	14	13	24.0	16/24	Ligaments Reconstructed	PCL, ACL collaterals
Ma W et al., 2023 [17]	Retrospective comparative study	70	48	22	60.3	17/24	PCL, LCL/PLC, MCL, ACL, in that order. In staged cohort. ACL was performed in subsequent stage.	NR
Mortazavi et al., 2022 [18]	Retrospective comparative study	18	11	7	24.0	16/24	Stage 1 Collateral ligament repair, stage II Cruciate reconstruction	PCL, ACL, LCL/PLC and MCL/PMC
Watrinet et al., 2022 [19]	Retrospective comparative study	38	20	15.0	66.0	14/24	PCL, ACL, LCL/PLC, and MCL/PMC	NR
Sun et al., 2016 [20]	Retrospective comparative study	32	21	11.0	34.7	18/24	NR	ACL PCL, PLC, PMC
Gan et al., 2023 [21]	Retrospective case series	47	45	0	53.2	12/16	ACL, PCL, PMC, PLC	PCL, ACL, Collaterals
He et al., 2024 [22]	Retrospective comparative study	102	102	0	87.6	14/24	PCL, ACL, Collaterals	ACL/PCL/LCL/MCL, PCL
Billières et al., 2020 [23]	Retrospective case series	20	20	0	29.4	11/16	ACL, PCL, MCL, LCL, PLC	NR
Hirschmann et al., 2010 [24]	Retrospective case series	24	24	0	72.0	14/16	ACL, PCL, MCL, LCL	PCL, ACL, Collaterals
Merle du Bourg et al., 2024 [25]	Retrospective case series	35	35	0	90.0	10/16	PCL, ACL, MCL, LCL	PCL, ACL, Collaterals
Arojuraye et al., 2025 [26]	Retrospective comparative study	51	26	25	12.0	18/24	PCL, ACL	PCL, ACL, MCL LCL
Ciancio et al., 2024 [27]	Prospective case series	18	18	0	24.0	11/16	PCL, ACL, MCL, LCL	PCL, ACL, Collaterals
Mussell et al., 2024 [28]	Retrospective case series	119	119	0	97.2	14/16	PCL, ACL then Collaterals	NR
Tardy et al., 2017 [29]	Retrospective cohort study	39	39	0	57.0	17/24	LCL, PCL, PCL ACL	ACL, PCL, PMC,
Hirschmann et al., 2010 [30]	Retrospective case series	74	74	0	144.0	14/16	ACL, PCL, PMC	PCL, ACL, Collaterals
Sanders et al., 2018 [31]	Retrospective case series	61.0	61	0	45.6	15/16	PCL, ACL, Collaterals	NR
Sundararajan et al., 2018 [32]	Prospective cohort study	45.0	45	0	36.0	17/24	ACL PCL Collaterals PLC	NR
Boos et al., 2024 [33]	Retrospective case series	55.0	55	0	182.0	13/16	ACL PCL Collaterals PLC	NR
Cain et al., 2024 [34]	Retrospective case series	53.0	53	0	92.4	14/16	ACL PCL Collaterals	NR
Pizza et al., 2023 [35]	Retrospective case series	42.0	42	0	115.2	12/16	ACL PCL Collaterals PLC	NR
Panish et al., 2024 [36]	Prospective case series	27	27	0	24.0	12/16	ACL PCL Collaterals PLC	ACL PCL Collaterals
Mhaskar et al., 2024 [37]	retrospective study	44	44	0	36.0	13/16	ACL PCL Collaterals	PCL, PLC, MCL ACL
Mayne et al., 2024 [38]	Prospective case series	40	40	0	24.0	14/16	ACL PCL Collaterals PLC	PCL, ACL, PLC, Collaterals
Said et al., 2023 [39]	Retrospective case series	75	75	0	78.0	13/16	PCL, ACL, PLC, Collaterals	PCL, ACL, Medial collateral, lateral
Korber et al., 2024 [40]	Retrospective case series	126	126	0	37.9	20/24	PCL, ACL, PLC, Collaterals	NR
Schneebeli et al. [41]	Retrospective + Prospective case series	52	52	0	45.6	12/16	PCL, ACL, PLC, Collaterals, PMC	PCL, Collaterals, ACL
Gupta et al., 2020 [42]	Retrospective case series	50	50	0	80.5	12/16	PCL, collaterals, ACL	PLC, Collaterals, ACL
Goyal et al., 2021 [43]	Retrospective case series	27	27	0	24.0	10/14	ACL/PCL	PCL, ACL, PLC, MCL
Cui et al., 2022 [44]	Prospective case series	17	0	17	11.18	14/16	PCL, ACL, PLC, MCL	PCL, ACL
Inada et al., 2021 [45]	Retrospective case series	25	0	25	24.8	11/16	MCL stage 1. PCL and ACL stage II	NR
Alentorn-Geli et al., 2019 [46]	Retrospective case series	39	39	0	27.0	12/16	PCL stage 1	NR
Ranger et al., 2018 [47]	Retrospective case series	111	111	0	74.4	10/24	PCL, collaterals, ACL	NR
Panigrahi et al., 2016 [48]	Retrospective case series	20	18	0	26.0	14/16	PCL, ACL, Collaterals, PMC, PLC	NR
Gliatis et al., 2018 [49]	Retrospective case series	31	31	0	60.0	8/16	PCL, ACL, LCL/PLC. MCL not repaired	PCL, ACL, LCL/PLC
Rusdi et al., 2014 [50]	Retrospective case series	21	21	0	21.7	9/16	PCL, PLC	NR
Khakha et al., 2016 [51]	Retrospective case series	36	36	0	121.0	10/16	PCL, ACL, MCL, LCL	NR
Hayash et al., 2008 [52]	Retrospective case series	19	19	0	42.0	6/16	PCL, ACL, MCL, LCL/PLV	PCL, ACL, MCL, PLC
Engebretsen et al., 2009 [53]	Retrospective case series	85	51	34	60.0	12/16	PCL, ACL, MCL, PCL/LCL	LC, PLC, PCL, ACL, MCL
Tao et al., 2013 [54]	Retrospective case series	9	9	0	30.0	10/16	LC, PLC, PCL, ACL MCL	PCL, ACL, PLC, MCL
Li et al. [55]	Retrospective case series	12	12	0	24.0	8/16	LC, PLC, PCL, ACL MCL	PCL, MCL, ACL, and LCL
Bagherifard et al., 2019 [56]	Retrospective case series	41	4	0	36.9	10/16	PCL, MCL, ACL, and LCL	PCL, PLC, ACL, and MCL
LaPrade et al., 2019 [57]	Retrospective case series	194	19	0	42.0	12/16	PCL, PLC, ACL, and MCL	NR
Bin et al., 2007 [58]	Retrospective case series	15	5	10	88.9	8/16	LCL, MCL stage 1, ACL and PCL stage 2	NR

**Table 3:** Functional scoring in single-stage and multi-stage surgeries.

Metric	Single-Stage (n = 1636)	Staged/Multi-Stage (n = 135)	Effect Size (95% CI)	*p*-Value
Lysholm Score	82.47 ± 11.32	87.21 ± 7.65	Mean difference: −4.74 [−6.33, −3.15]	<0.001
IKDC Score	76.31 ± 15.25	77.88 ± 9.45	Mean difference: −1.57 [−4.23, 1.10]	0.19

**Table 4:** Complication rates.

Complication	Single-Stage Rate	Multi-Stage Rate	Odds Ratio (95% CI)	*p*-Value
Arthrofibrosis	7.29%	1.95%	3.96 (1.25–12.57)	0.007
Graft Failure	3.08%	0.65%	4.85 (0.67–35.31)	0.125
Infection	0.84%	1.95%	0.42 (0.12–1.49)	0.167

## Data Availability

Template data collection forms, extracted study-level data, and analytic code are available upon reasonable request from the corresponding author. Due to data privacy considerations, these materials have not been made publicly available online.

## Abbreviations

The following abbreviations are used in this manuscript:

ACL	anterior cruciate ligament
BMI	body mass index
IKDC	International Knee Documentation Committee
LARS	Ligament Advanced Reinforcement System
LCL	lateral collateral ligament
MCL	medial collateral ligament
MD	mean difference
MINORS	Methodological Index for Non-Randomized Studies
MLKI	multi-ligament knee injury
MUA	manipulation under anesthesia
PCL	posterior cruciate ligament
PLC	posterolateral corner
PRISMA	Preferred Reporting Items for Systematic Reviews and Meta-Analyses
ROM	range of motion
RTS	return to sport
SE	standard error
SMD	standardized mean difference
